# Genetic characterization of inbred lines from Shaan A and B groups for identifying loci associated with maize grain yield

**DOI:** 10.1186/s12863-018-0669-9

**Published:** 2018-08-23

**Authors:** Ting Li, Jianzhou Qu, Yahui Wang, Liguo Chang, Kunhui He, Dongwei Guo, Xinghua Zhang, Shutu Xu, Jiquan Xue

**Affiliations:** 1 Key Laboratory of Biology and Genetic Improvement of Maize in Arid Area of Northwest Region, Ministry of Agriculture, College of Agronomy, Northwest A&F University, Yangling, 712100 Shaanxi China; 2 Maize Engineering Technology Research Centre of Shaanxi Province, Yangling, China

**Keywords:** Maize, Genetic diversity, Grain yield, Genome-wide association study

## Abstract

**Background:**

Increasing grain yield is a primary objective of maize breeding. Dissecting the genetic architecture of grain yield furthers genetic improvements to increase yield. Presented here is an association panel composed of 126 maize inbreds (AM126), which were genotyped by the genotyping-by-sequencing (tGBS) method. We performed genetic characterization and association analysis related to grain yield in the association panel.

**Results:**

In total, 46,046 SNPs with a minor allele frequency (MAF) ≥0.01 were used to assess genetic diversity and kinship in AM126. The results showed that the average MAF and polymorphism information content (PIC) were 0.164 and 0.198, respectively. The Shaan B group, with 11,284 unique SNPs, exhibited greater genetic diversity than did the Shaan A group, with 2644 SNPs. The 61.82% kinship coefficient in AM126 was equal to 0, and only 0.15% of that percentage was greater than 0.7. A total of 31,983 SNPs with MAF ≥0.05 were used to characterize population structure, LD decay and association mapping. Population structure analysis suggested that AM126 can be divided into 6 subgroups, which is consistent with breeding experience and pedigree information. The LD decay distance in AM126 was 150 kb. A total of 51 significant SNPs associated with grain yield were identified at *P* < 1 × 10^− 3^ across two environments (Yangling and Yulin). Among those SNPs, two loci displayed overlapping regions in the two environments. Finally, 30 candidate genes were found to be associated with grain yield.

**Conclusions:**

These results contribute to the genetic characterization of this breeding population, which serves as a reference for hybrid breeding and population improvement, and demonstrate the genetic architecture of maize grain yield, potentially facilitating genetic improvement.

**Electronic supplementary material:**

The online version of this article (10.1186/s12863-018-0669-9) contains supplementary material, which is available to authorized users.

## Background

Maize (*Zea mays* L.), the most widely grown crop in the world, plays an essential role in global food security and industrial products [1]. As a cross-pollinated crop, genomic divergence is nearly 1.42% between two maize inbred lines, which is greater than the divergence of 1.34% between humans and chimpanzees [2]. This great genomic diversity has resulted in considerable phenotypic variety. Moreover, maize is an important model plant for studying genome evolution, heterosis and the genetic architecture of complex quantitative traits [3]. According to statistical data from the FAO, the predicted worldwide population of 9 billion by 2050 will require 70% more food than today’s population [4]. It is estimated that more than half of the increased demand for cereals will come from maize, which is the crop with the largest planted area and highest total production. The necessary increase in maize production will require substantial changes in agronomic practices and methods of genetic improvement [5]. Previously, yield improvement has occurred at the expense of environmental pollution from increased fertilizer use [6, 7]. Along with the increasing focus on green production, more work has been aimed at increasing yield through genetic improvements, through which several QTLs and genes associated with grain yield and yield-related traits had been validated. For example, five QTLs showing a high genetic relationship with the phenotypic variance of yield component traits [8] and one large-effect QTL influencing kernel row number located on chromosome 7 was identified using linkage mapping [9]. Additionally, a stable locus related to kernel shape, PKS2, was identified through linkage and association analysis in 240 maize inbreds [1].

In the last several decades, the power and resolution of QTL mapping for complex quantitative traits, such as flowering, drought resistance, the contents of fatty acids and minor elements (carotenoids and tocopherols), metabolome features and kernel rows, has greatly increased because of the development of association analyses, including candidate gene association mapping and GWAS in maize and other species [10–12]. However, the increasingly wide application of association mapping is due to the rapid development of genotyping techniques, which has produced effective high-throughput molecular technology. A kind of molecular markers were developed by different genotyping technologies, among SSR makers were used to evaluate the polymorphisms of *Dwarf 8* associated with flowering time in maize in the early days of development [13]. Later, association analyses using SSR markers were performed in plants to dissect complex quantitative traits [14, 15]. At present, SNP markers are widely utilized in association analyses of spring wheat, rice, *Arabidopsis thaliana* and maize [16–19], because of the advantages of biallelic markers and their higher content in the genome. Large numbers of these markers have been exploited through SNP chip and genome sequencing technology.

For maize, a variety of SNP chips based on SNP genotyping platforms have been designed by sequencing known genes for genotyping. These chips include the Illumina® SNP1536 chip, the MaizeSNP50 BeadChip with a high resolution, and a higher-density 600 k SNP genotyping array based on 57 M SNPs and small indels determined from 30 representative temperate maize lines in comparison with B73 AGP_v3 [20–22]. However, using SNP chip analysis as a genotyping method is expensive and fixed. In addition, the ability to detect ectopic exchange points is very weak. In comparison, genotyping-by-sequencing (GBS) is a recently developed simple sequencing procedure that can provide a large number of markers across the genome at low cost per sample and can be applied to maize, which exhibits high diversity and a large genome [23]. The GBS method does not rely on previous knowledge of SNPs and greatly expands the number of individuals and markers that can be studied, which increases the chance of discovering more uncommon or rare variants [24, 25].

Increasing grain yield is the primary target for meeting the food demand of the growing population, and dissecting the genetic architecture of grain yield is helpful for achieving this goal. Due to the complexity of the genetic architecture of grain yield and the difference between association populations, some researchers have aimed to uncover the genetic architecture of grain yield through association mapping; however, these studies are far from sufficient. Therefore, in this study, 126 maize inbred lines from the Shaan A and Shaan B groups were selected for genotyping with tGBS sequencing technology. Our aims were 1) to perform detailed characterization of the association mapping panel, including relationships, population structure, and genetic diversity; and 2) to dissect the genetic basis of grain yield in the association mapping panel.

## Methods

### Plant materials and field experiments

The association mapping panel consisted of 126 diverse inbred lines (AM126) selected from Shaan A and Shaan B group inbreds cultivated at Northwest A&F University. According to the theory of domestic and international maize breeding, we simplified heterotic model and adopted the breeding strategy of two divergent heterotic groups to build Shaan A and Shaan B heterotic groups, in which superior varieties are employed as basic materials to adapt maize production to Shaanxi Province [26]. High-density planting, drought, low fertilizer use and multiple environments were carried out to the expansion, improvement and utilization of the germplasm. From 2007 to 2008, basic groups were constructed over three generations. From 2009 to 2015, the Shaan A group and Shaan B group were optimized and upgraded through 7 rounds of selection in 30 departments in seven provinces (Shaanxi, Gansu, Henan, Hebei, Neimenggu, Sichuan and Xinjiang). Ultimately, we explored a technical approach for continuous improvement of maize germplasm and successfully built the Shaan A and Shaan B groups. In AM126, 94 different inbred lines belonged to the Shaan B group, and the others belonged to the Shaan A group. Detailed information about the 126 inbred lines is provided in Additional file 1. These inbred lines were planted in Yangling (34°16′N, 108°40′E) and Yulin (38°30′N, 109°77′E) in Shaanxi Province in 2017. At each location, all inbred lines were planted in a two-row plot using a randomized experimental design with two replications, with a row length of 5 m and a distance of 0.6 m between adjacent rows. The planting density was 67,500 plants/ha. During growth, field management followed normal field operations.

### Phenotypic evaluation and analysis

Upon harvest, all ears were harvested by hand threshing, and corresponding data, including the grain water content, total grain weight and weight of ten panicles, were recorded to calculate the grain yield per mu (kg) by multiplying the grain yield per panicle by the total number of plants in the plot and adjusting to a 14% moisture content. Then, the mean grain yield of two replicates was calculated for subsequent analysis (Additional file 2).

Phenotypic data analyses, which included basic descriptive statistical analyses, ANOVA and Pearson correlation analysis, were carried out using SPSS v.22 software (IBM crop. Armonk, NY, USA). According to the method described by Knapp et al. [27], the broad-sense heritability (*h*^*2*^) of yield is estimated with the formula: ${h}^2={\upsigma}_{\mathrm{g}}^2/\left({\upsigma}_{\mathrm{g}}^2+{\upsigma}_{\mathrm{g}\mathrm{e}}^2/\mathrm{n}+{\upsigma}_3^2/n\mathrm{k}\right)$, where σ_g_^2^ is the genetic variance; σ_ge_^2^ is the interaction variance between the genotype and environment; σ_з_^2^ represents the residual error variance; and n and k represent the environment and number of replications, respectively.

### Genotyping

Total genomic DNA was extracted from leaf samples of each inbred line based on the CTAB procedure [28]. Fundamental qualities were evaluated by gel electrophoresis and spectrophotometry (Nanodrop2000, Thermo Scientific) in our laboratory. More stringent DNA quality testing and sequencing were completed by Data2Bio (D2B; LLC, Ames, IA, USA). The tGBS protocols followed by Data2Bio were described previously [29]. Briefly, 299,598,955 raw reads were generated from the 126 maize samples through six Ion Proton runs. Prior to alignment, the nucleotides of each raw read were scanned for low-quality bases. Bases with PHRED quality scores of < 15 out of 40 (≤3% error rate) were trimmed [30, 31]. Subsequently, the trimmed reads from each sample were aligned to GenomeB73_RefGenV4 using GSNAP [32], and confidently mapped reads were filtered if they mapped uniquely (≤2 mismatches every 36 bp and < 5 bases for every 75 bp as tails). Finally, 46,046 SNPs were filtered according to the following criteria (Additional file 3): 1. minimum calling rate ≥ 50%; 2. minor allele frequency (MAF) ≥0.01; 3. allele number = 2; 4. genotype ≥2; and 5. heterozygosity rate of 0% ~ (2 x Frequency_allele1_ x Frequency_allele2_ + 20%) from the TASSEL-GBS Pipeline [33].

### Genetic diversity

The polymorphism information content (PIC) and MAF, which can be used to evaluate the genetic diversity of the population, were calculated with Powermarker v3.25 [34] using 46,046 SNPs. The PIC can reflect the degree of DNA mutation in a population and can be estimated as follows:

$$ {\overset{\hat{\mkern6mu} }{\mathrm{P}\mathrm{IC}}}_{\mathrm{l}}=1-\sum \limits_{\mathrm{u}=1}^{\mathrm{k}}{\overset{\sim }{\mathrm{P}}}_{\mathrm{l}\mathrm{u}}^2-\sum \limits_{\mathrm{u}=1}^{\mathrm{k}-1}\sum \limits_{\mathrm{v}=\mathrm{u}+1}^{\mathrm{k}}2{\overset{\sim }{\mathrm{P}}}_{\mathrm{l}\mathrm{u}}^2{\overset{\sim }{\mathrm{P}}}_{\mathrm{l}\mathrm{v}}^2 $$

where Plu and Plv refer to the frequency of the uth and vth alleles of marker l, respectively. A PIC value from 0 to 0.25 indicates low polymorphism; a PIC value from 0.25 to 0.5 indicates intermediate polymorphism; and a PIC value from 0.5 to 1 indicates high polymorphism. The MAF was used to quantify the degree of genetic differentiation in the maize population. The ratio of the number of SNPs with less variation to the total number of SNPs at each SNP locus was calculated. To avoid the influence of sample size, a re-sampling strategy was adopted in this study. The distribution of the SNPs unique to the Shaan A inbred lines or the Shaan B group inbred lines on ten chromosomes was determined with the ggplot R package.

### Population structure and relative relationships

The relative kinship matrix between inbred lines i and j was calculated with TASSEL v.5.0 software to explore the pairwise relationships of the 126 inbred lines. The results were illustrated with the Genomic Association and Prediction Integrated Tool-R (GAPIT) package [35]. All negative values between pairwise lines were set to 0 [36].

In addition, to rapidly investigate population structure, 31,983 high-quality SNPs were screened with stringent criteria (missing rate ≤ 0.05, MAF ≥0.05) using TASSEL v.5.0 software and imported into Admixture software version 1.23 for cross validation [37]. The optimal partitioning of subgroups (K) was determined from the mixed cross validation error values, which were computed from the number of subpopulations (K), ranging from 1 to 15. The output of the Admixture software was imported into R to create a stacked bar chart.

### SNP-based genome-wide association mapping and gene annotation

A total of 31,983 SNPs with MAF ≥0.05 and missing rate ≤ 50% were filtered for the genome-wide association study (GWAS). The GWAS of the grain yield data from the two locations was accomplished in the TASSEL v.5.0 software with a mixed linear model (MLM), controlling for population structure and relative kinship (K + Q) to avoid spurious associations [33]. When using the Bonferroni correction threshold for GWAS, we found no significant association between the grain yields from the two locations. Therefore, *P* < 1 × 10^− 3^ was chosen to determine significant SNPs for the trait. Thereafter, the LD decay distance in AM126 was estimated using TASSEL v.5.0 software. Finally, we confirmed the unique candidate genes underlying the association signals with SNP markers based on the LD decay distance of this population and annotated the candidate genes according to the information available in the Maize Sequence (http://ensembl.gramene.org/Zea_mays/Info/Index) and the MaizeGDB (http://www.maizegdb.org/gbrowse) databases. Because only a version 3 gene annotation file exists, all v4 gene IDs were converted to v3 gene IDs and then annotated.

## Results

### Basic SNP statistics of AM126 based on tGBS sequencing

Through tGBS sequencing, 299,598,955 raw reads were generated from AM126 and uniquely aligned to the reference genome (http://ensembl.gramene.org/Zea_mays/Info/Index, AGPV4). Ultimately, 1,133,188 sites were identified, among which 46,046 SNPs were polymorphic in AM126, with a missing rate of less than 50% and MAF of more than 0.01. For the 46,046 SNPs, the number of SNPs per chromosome ranged from 3235 SNPs on chromosome 10 to 6513 SNPs on chromosome 1 (Table 1). Chromosome 3 showed the lowest average marker density and chromosome 5 the greatest. The average marker density across the ten chromosomes was found to be approximately 45.7 kb. For the 31,983 high-quality SNPs with an MAF ≥0.05, the number of SNPs per chromosome ranged from 2287 SNPs on chromosome 10 to 4494 SNPs on chromosome 1. The average distance between neighbouring markers on different chromosomes varied from 59.4 to 69.8 kb, with an average of approximately 65.9 kb. The proportion of the reduction of SNPs with MAF < 0.05 was greater on chromosomes 1, 6 and 7 than on the other chromosomes.

**Table 1:** Chromosomal distribution and proportion of polymorphic markers used for computing genetic diversity and relationships (46,046 SNPs) and for population structure, LD decay and association analyses (31,983 SNPs)

Chromosome	46,046 SNPs			31,983 SNPs		
	No. of markers	Proportion	Marker density (kb)	No. of markers	Proportion	Marker density (kb)
1	6513	14.14%	47.1	4494	14.05%	68.3
2	5120	11.12%	47.8	3560	11.13%	68.7
3	5692	12.36%	41.4	3967	12.40%	59.4
4	5513	11.97%	44.8	3857	12.06%	64.0
5	4678	10.16%	47.9	3284	10.27%	68.2
6	3684	8.00%	47.2	2492	7.79%	69.8
7	3956	8.59%	46.1	2651	8.29%	68.8
8	4110	8.93%	44.1	2863	8.95%	63.3
9	3545	7.70%	45.1	2528	7.90%	63.2
10	3235	7.03%	46.7	2287	7.15%	66.0
Average	4604.6	10.00%	45.7	3198.3	10.00%	65.9

### Genetic diversity

**Fig. 1:**
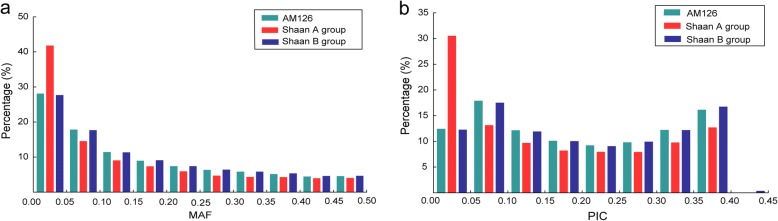
Distribution of MAF and PIC in AM126, the Shaan A group and the Shaan B group. MAF distribution (**a**) and PIC distribution (**b**)

A total of 46,046 SNPs were used to estimate the MAF and PIC for AM126 and each group. The MAF and PIC distribution of all SNPs are provided in Fig. 1. Among the 46,046 SNPs, 28.08% showed an MAF of less than 0.05, and 12.43% exhibited a PIC of less than 0.05 in AM126. The average MAF for AM126 was 0.164, varying from 0.010 to 0.500, and the average PIC was 0.198, varying from 0.020 to 0.398 (Table 2). The Shaan B group showed a higher average MAF (0.166) and PIC (0.200) than the Shaan A group, which displayed an average MAF of 0.134 and PIC of 0.157. Furthermore, 32 inbred lines (the same sample size as for Shaan A) were selected randomly from the Shaan B group 10 times to eliminate the effect of sample size. The results confirmed that the Shaan B group exhibited higher genetic diversity than the Shaan A group, with an average MAF of 0.161 (0.154–0.178) and PIC of 0.191 (0.180–0.220).

**Table 2:** MAF and PIC of different groups determined using 46,046 SNPs

Group	No. of lines	MAF	PIC
AM126	126	0.164 (0.010–0.500)	0.198 (0.020–0.398)
Shaan A group	32	0.134 (0.000–0.500)	0.157 (0.000–0.375)
Shaan B group	94	0.166 (0.000–0.500)	0.200 (0.000–0.409)
Shaan B group (re-sampled)	32	0.161(0.154–0.178)	0.191(0.180–0.220)

**Fig. 2:**
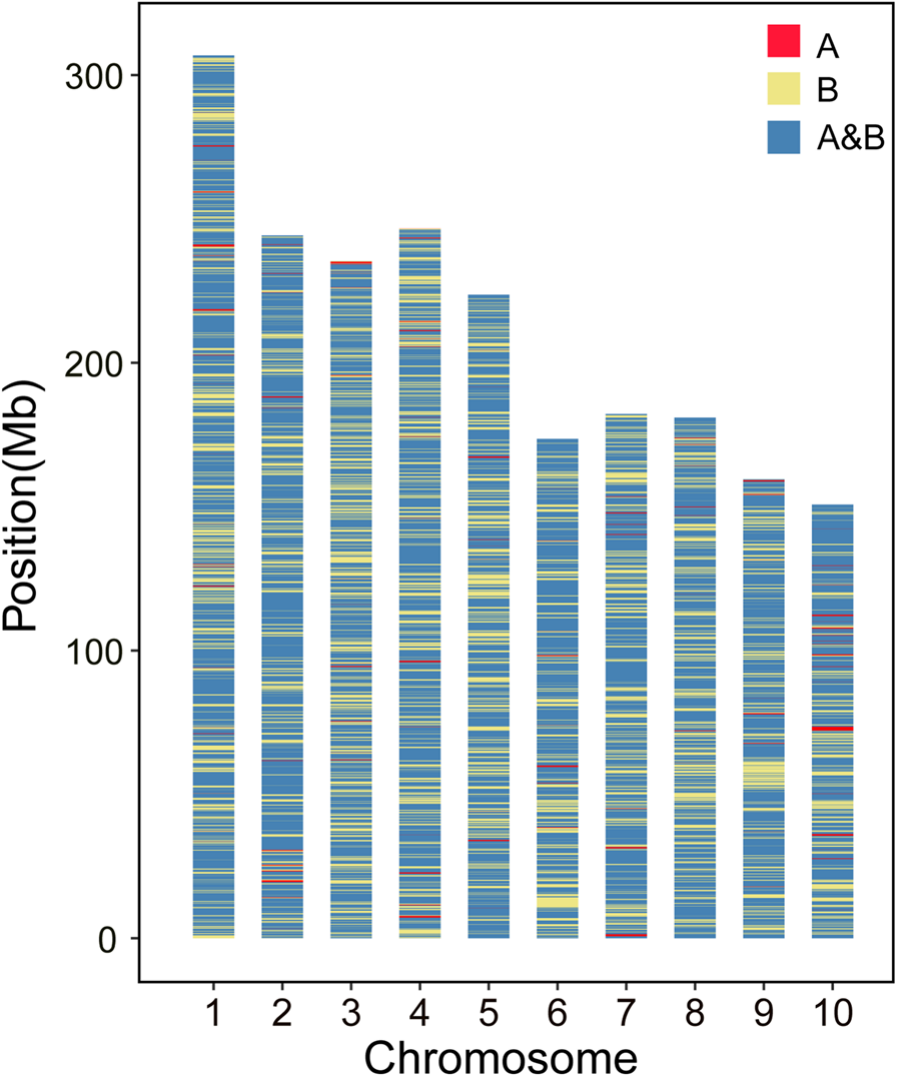
Distribution of polymorphic loci across the genome in the Shaan A and Shaan B groups. The distribution of unique SNPs in the Shaan A and Shaan B groups is labelled in red and yellow, respectively, and blue represents the SNPs common to the Shaan A and Shaan B groups

The chromosomal distribution of unique polymorphic sites is provided for further comparison of the genetic diversity between the Shaan A and Shaan B groups (Fig. 2). Among the 46,046 SNPs, 11,284 SNPs (24.5%) were unique to the Shaan B group and were widely distributed on all chromosomes, whereas the Shaan A group contained 2644 unique SNPs, which only accounted for 5.7% of the total. The number and distribution of unique SNPs on chromosomes showed obvious differences between the Shaan A and Shaan B groups. The analysis indicated that Shaan B had a broader genetic basis than Shaan A group.

### Relative kinship and population structure

**Fig. 3:**
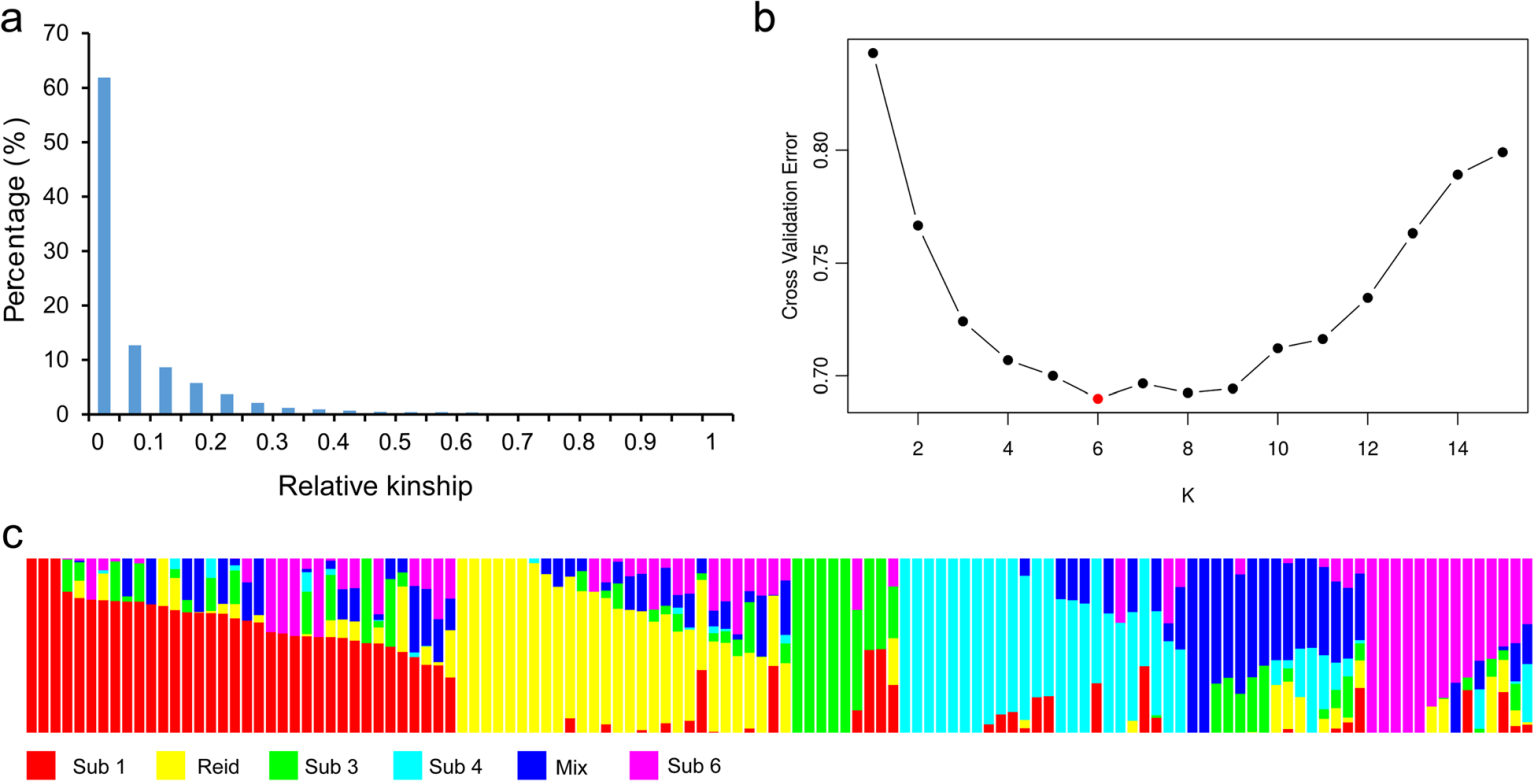
Distribution of the pairwise kinship and population structure in AM126. The proportion of pairwise kinship coefficients ranging from 0 to 1 is shown (**a**). Plot of the cross validation error value in the range of K = 1 to 15 (**b**). Population structure based on k = 6 (**c**)

To elucidate the relationships among the inbred lines, all 46,046 SNPs were used to compute kinship coefficients. The pairwise relative kinship coefficients in AM126 ranged from 0.00 to 1.03. A total of 61.82% of the relative kinship values were equal to 0, and 36.62% of the relative kinship values varied from 0.05 to 0.5. Only 0.15% of the relative kinship values exceeded 0.7. The remaining 1.38% of the paired relative kinship values ranged from 0.5 to 0.7 (Fig. 3a, Additional file 4). The kinship heatmap is shown in Additional file 5. Low relative kinship was observed for AM126, which is consistent with known pedigrees.

The 31,983 high-quality SNPs were used to estimate ancestry in Admixture, based on the maximum-likelihood approach [37]. The cross validation error value for K ranging from 1 to 15 was computed to infer the population structure of AM126 (Fig. 3b). The lowest cross validation error value was observed when K = 6, suggesting that AM126 could be divided into six subgroups (Subs 1, 2, 3, 4, 5, and 6) (Fig. 3c, Additional file 6). In addition, comparison with previous breeding experience and pedigree backgrounds also indicated that K = 6 was a logical number for the subpopulation. PH6WC, belonging to the Reid group, was included in Sub 2, which was composed of 4 inbreds selected from the Shaan B group and 24 inbreds selected from the Shaan A group. Therefore, Sub 2 is also referred to as the Reid subgroup. Sub 1 contained 33 inbreds from the Shaan B group and 3 inbreds from the Shaan A group. Sub 3 consisted of 9 inbreds from the Shaan B group, which was a much smaller number than in the other subpopulations. Twenty-four Shaan B group inbred lines were clustered into Sub 4. Twelve Shaan B inbred lines and 2 Shaan A inbred lines were grouped into Sub 6. Additionally, 3 Shaan A inbred lines and 12 Shaan B inbred lines that showed a lower probability were assigned to Sub 5, which was also referred to as the mix subgroup. The resulting population structure of AM126 can be used for further analysis and shows that the Shaan A group presents less ancestral diversity than the Shaan B group.

### Genome-wide association study

The grain yield was counted and was found to follow a normal distribution at each location (Additional file 7). In Yangling and Yulin, the average yields were 195.11 and 442.26 kg/mu, varying from 81.59 to 338.36 and 282.45 to 687.58 kg/mu, respectively (Table 3), and the coefficient of variation (CV) were 25.00% and 17.39%, respectively. The grain yields in Yangling and Yulin were significantly positively related at the *p* = 0.01 level, with a Pearson correlation coefficient of 0.519. Additionally, in this panel, the grain yield displayed a high heritability of over 83.33%. These results suggested that the grain yield was highly variable in this population.

**Table 3:** Descriptive statistics, correlation coefficient between the two environments and broad-sense heritability of the yield traits

Environment	Mean ± SD	Range	CV(%)	Correlation coefficient	*h*2 (%)
				Yang ling	
Yangling	195.11 ± 48.77	81.59–338.36	25.00		83.33
Yulin	442.26 ± 76.93	282.45–687.58	17.39	0.519^a^	

^a^Significant different at 0.01 level

**Fig. 4:**
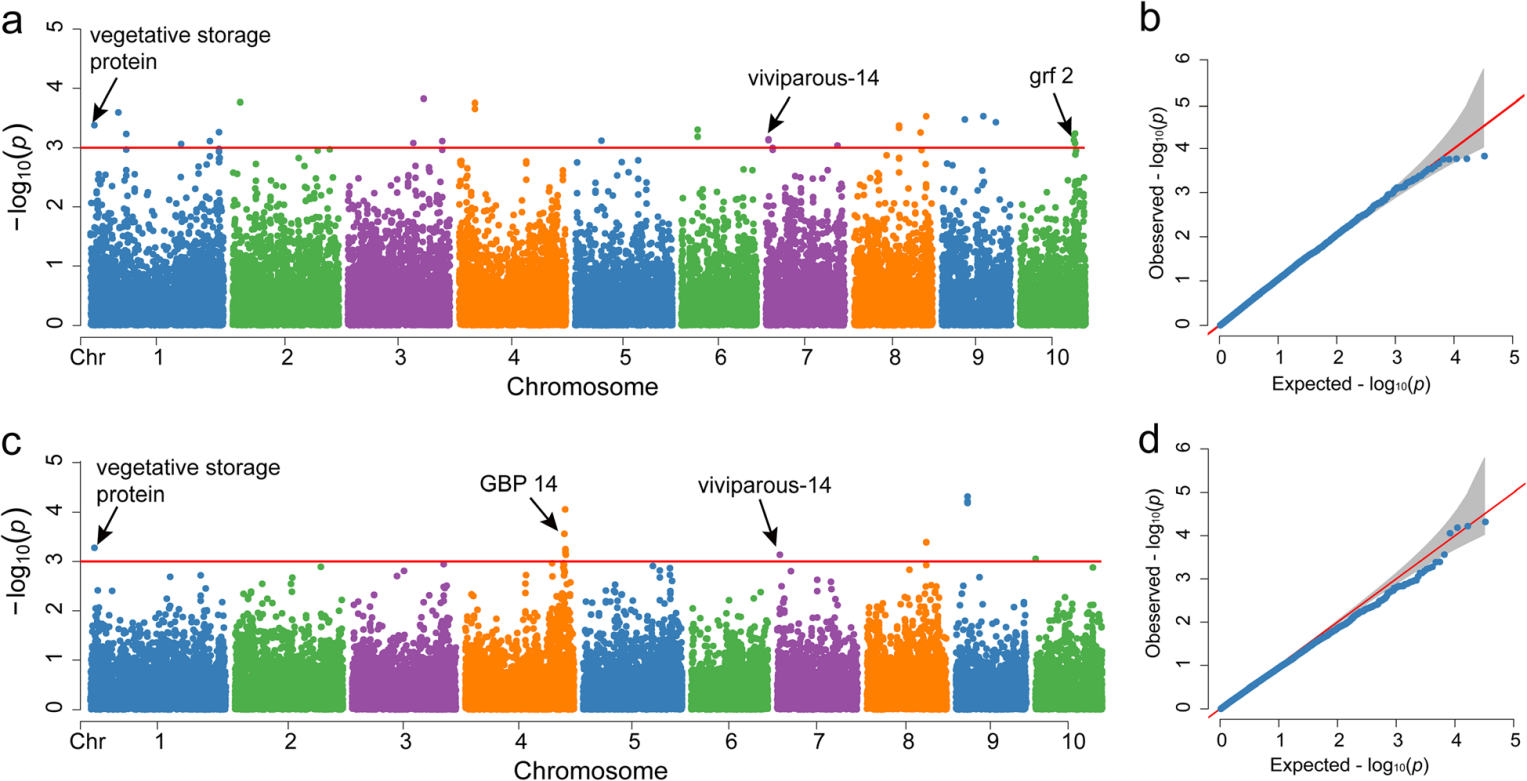
Results of the GWAS of grain yield in AM126. Manhattan plot (**a**) and quantile-quantile plot (**b**) in Yangling. Manhattan plot (**c**) and quantile-quantile plot (**d**) in Yulin. The red line in the Manhattan plots corresponds to the Bonferroni-adjusted threshold (*P* < 1 × 10^− 3^)

GWAS of the grain yield of AM126 was performed separately for the two locations (Yangling and Yulin) using 31,983 high-quality SNPs. As shown in the quantile-quantile plots of grain yield (Fig. 4b, d), fewer false positives were found after application of the MLM with a population structure and relationship (Q + K) model, and we used these results to annotate associated genes and identify candidate genes. A total of 51 lead SNPs, corresponding to 33 loci, were significantly associated (*P* < 1 × 10^− 3^) with yield. Only one of these loci was the same in the two environments. The Manhattan plots showed that the SNPs associated with yield were spread across the genome in Yangling and were distributed mainly on chromosome 4 in Yulin (Fig. 4a, c). When the R^2^ value was less than 0.01, the LD decay distance was about 150 kb in AM126 (Additional file 8). Therefore, we identified candidate genes in a 300 kb region around the positions of significantly associated SNPs and discovered that two intervals in the samples from the two environments were mostly overlapping.

**Fig. 5:**
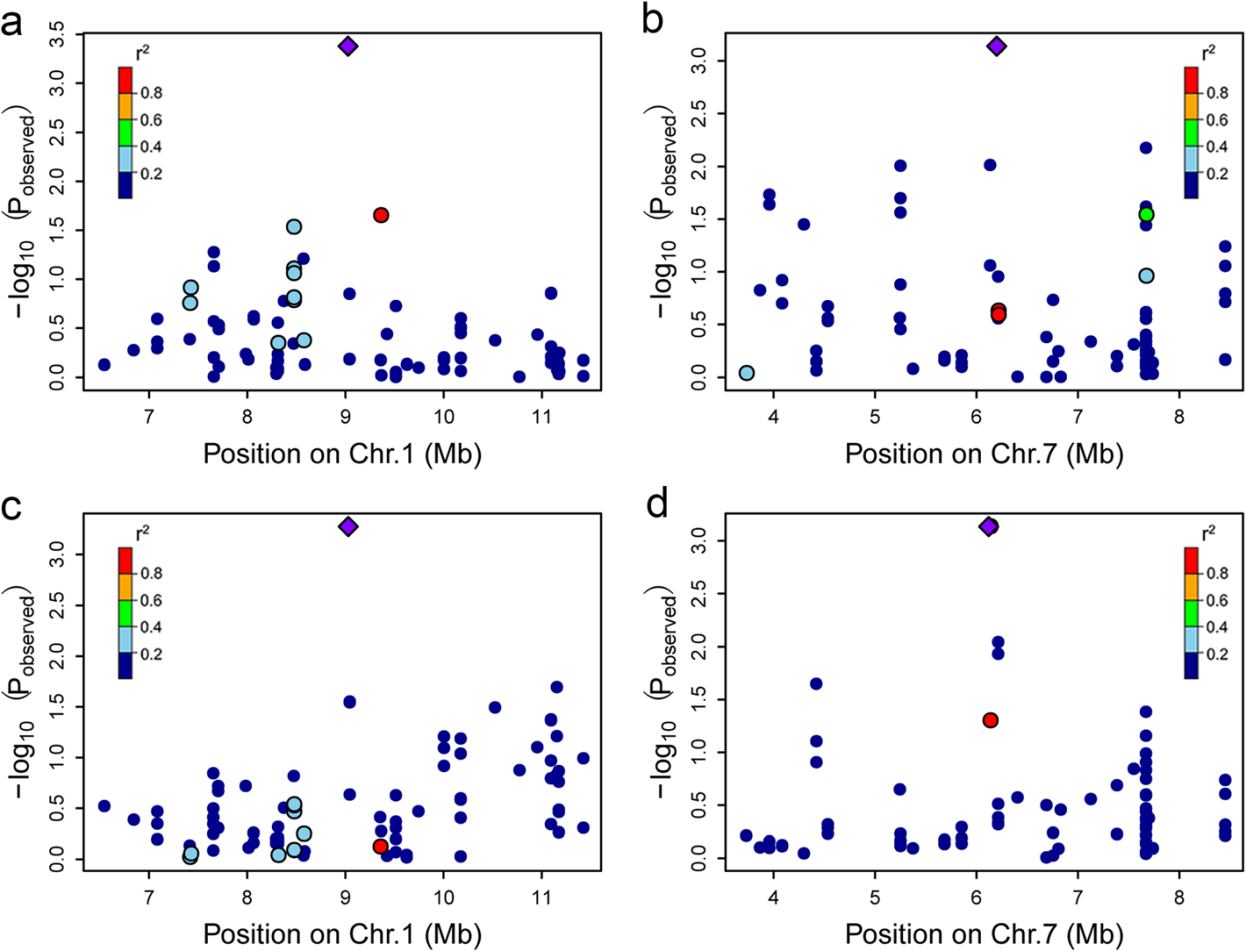
Region plot of four SNPs associated with grain yield, which are located within 2.5 Mb on both sides of the lead SNP. Zm00001d027610 was identified based on the lead SNP, which was associated with grain yield in Yangling (**a**) and Yulin (**c**). Zm00001d018819 was identified based on the lead SNP, which was related to grain yield in Yangling (**b**) and Yulin (**d**)

According to the gene and protein annotations from Maize Sequence (http://ensembl.gramene.org/Zea_mays/Info/Index), MaizeGDB (http://www.maizegdb.org) and InterPro (http://www.ebi.ac.uk/interpro), genes that may be associated with yield were identified and were illustrated in Table 4. Among the genes with functional annotations, Zm00001d027610, which encodes a vegetative storage protein and is located within the overlapping interval on chromosome 1 (8,876,216-9,176,216 bp), appeared to be a candidate gene that may be associated with grain yield (Fig. 5a, c). In another region with overlapping interval on chromosome 7 (5,969,535-6,348,940 bp), we identified the gene Zm00001d018819, encoding viviparous-14, which is involved in the abscisic acid (ABA) biosynthesis pathway (Fig. 5b, d). In addition, 28 candidate genes were identified based on significant SNPs associated with grain yield in a single environment. Zm00001d025617 encodes general regulatory factor (GF) 2, which belongs to the 14–3-3 family (IPR000308), on chromosome 10. Zm00001d053298, also known as *GBP14*, encodes the GLABROUS1 enhancer-binding protein (GeBP) transcription factor, which belongs to the GeBP family (IPR007592) and is located on chromosome 4. However, we did not find genes of known function at the loci with the lowest *p* values; these genes may not be directly involved in the relevant pathway, or the SNP loci may be linked with nearby genes.

**Table 4:** Markers and genes significantly associated with yield in the two environments

Environment	Chr	Pos	*P*-value	Marker R^2^	MAF	Candidate interval	Gene ID
Yangling	1	64,203,074	2.54E-04	0.182	0.071	64,053,074–64,353,074	Zm00001d029264
Yangling, Yulin	1	9,026,216	4.18E-04	0.149	0.107	8,876,216–9,176,216	Zm00001d027610
Yangling	1	296,366,071	5.48E-04	0.150	0.28	296,216,071–296,516,071	Zm00001d034563
Yangling	1	82,414,042	5.88E-04	0.179	0.071	82,264,042–82,564,042	Zm00001d029679
Yangling	1	275,324,785	7.76E-04	0.216	0.269	275,174,785–275,474,785	Zm00001d033834
Yangling	1	208,984,720	8.68E-04	0.113	0.061	208,834,720–209,134,720	Zm00001d031996
Yangling	2	17,192,443	1.72E-04	0.136	0.084	17,042,443–17,342,443	Zm00001d002623
Yangling	3	174,894,572	1.50E-04	0.174	0.255	174,744,572–175,044,572	Zm00001d042637
Yangling	3	217,771,512	7.76E-04	0.109	0.364	217,621,512–217,921,512	Zm00001d044048
Yangling	3	151,015,584	8.38E-04	0.110	0.054	150,865,319–151,165,584	Zm00001d042108
Yulin	4	226,628,381	8.83E-05	0.286	0.262	226,478,381–226,778,381	Zm00001d053334
Yangling	4	36,611,473	1.77E-04	0.182	0.349	36,461,473–36,761,473	Zm00001d049590
Yulin	4	224,904,708	2.76E-04	0.115	0.09	224,754,708–225,054,708	Zm00001d053298
Yulin	4	227,248,589	5.65E-04	0.133	0.248	227,098,589–227,398,589	Zm00001d053354
Yulin	4	228,002,806	7.35E-04	0.14	0.095	227,812,359–228,152,806	Zm00001d053369
Yulin	4	197,048,390	1.09E-03	0.116	0.232	196,898,390–197,198,390	Zm00001d052678
Yulin	4	222,979,053	1.09E-03	0.117	0.200	222,829,053–223,129,053	Zm00001d053259
Yangling	5	60,500,183	7.64E-04	0.102	0.064	60,350,183–60,650,183	Zm00001d014722
Yangling	6	37,566,120	4.97E-04	0.130	0.257	37,416,120–37,716,120	Zm00001d035629
Yangling	7	6,198,940	7.25E-04	0.140	0.285	5,969,535–6,348,940	Zm00001d018819
Yulin	7	6,119,535	7.34E-04	0.126	0.366	5,969,535–6,348,940	Zm00001d018819
Yangling	7	165,187,310	9.21E-04	0.113	0.087	165,037,310–165,337,310	Zm00001d021877
Yangling	8	166,364,288	2.95E-04	0.154	0.070	166,214,288–166,514,288	Zm00001d012007
Yulin	8	134,765,776	4.07E-04	0.226	0.176	134,615,776–134,915,776	Zm00001d010946
Yangling	8	103,836,414	4.24E-04	0.104	0.096	103,686,414–103,986,414	Zm00001d010201
Yangling	8	153,275,644	5.55E-04	0.129	0.133	153,108,098–153,425,644	Zm00001d011515
Yulin	9	26,330,295	6.05E-05	0.145	0.073	26,180,295–26,480,295	unknown
Yangling	9	95,895,364	2.94E-04	0.251	0.362	95,745,364–96,045,364	Zm00001d046558
Yangling	9	53,338,849	3.34E-04	0.125	0.105	53,188,849–53,488,849	Zm00001d046004
Yangling	9	124,887,914	3.72E-04	0.127	0.056	124,737,914–125,037,914	Zm00001d047266
Yangling	10	126,844,985	5.79E-04	0.121	0.056	126,694,985–126,994,985	Zm00001d025703
Yangling	10	123,993,567	6.10E-04	0.114	0.329	123,843,567–124,143,567	Zm00001d025617
Yulin	10	13,050	8.89E-04	0.100	0.154	0–163,050	unknown

## Discussion

Many researchers have analysed complex quantitative traits using association panels collected from hundreds of inbreds from all over the world, especially in maize [38, 39]. These inbreds usually originate from different breeding programmes around the world. However, the materials employed in present study were derived from the same breeding project, and they had been cultivated under high-stress conditions for nearly 10 years. Therefore, to analyse genetic diversity and kinship within this germplasm, the inbreds must be used more accurately in breeding programmes. Using the AM126 panel to perform association mapping will provide new insight into combining molecular genetics and conventional breeding.

Maize has abundant genetic diversity, and abundant genetic diversity of maize germplasm greatly benefits crop breeding [40]. Artificial selection for favourable alleles has gradually reduced genetic diversity in maize and increased the abundance of some favourable low-frequency alleles [41]. This selection will help us to identify new genes. In this study, the PIC and MAF of the entire panel were 0.198 and 0.164, respectively. These values are significantly lower than those reported in other studies of diversity in maize inbred lines [20], including the PIC and MAF of 0.29 and 0.33, respectively, obtained in a previous study using 2846 SNPs across 32 inbred lines selected from the Shaan A and Shaan B groups [42]. However, the average MAF was relatively higher than that of 538 maize inbred lines (CMLs) determined using 955,120 SNPs with MAF ≥0.01 [43]. These differences were mainly caused by the choice of maize germplasm and SNP filtration criteria. In general, compared to ordinary association mapping, the genetic diversity of the breeding population was lower. Additionally, GBS can produce a very high miss rate and a large number of SNPs with a very low frequency [44]. Low-frequency SNPs may facilitate the identification of complex traits that rely on low-frequency and rare variants [45]. In addition, the inbreds from the Shaan B group were found to be more diverse than those from the Shaan A group in this study. These results showed that inbreds from the Shaan A group might have experienced stricter selective conditions than those from the Shaan B group during inbred selection under the same environment.

Population structure is the foundation of hybrid breeding and a key factor in association mapping [36]. According to long-term breeding experience, maize inbreds have been divided into several heterotic groups. However, it remains unclear how many heterotic groups of maize exist; indeed, researchers have not reached a consensus in this regard. For maize in the USA, the classic view is that maize should be separated into two heterotic groups—stiff stalk (SS) and non-stiff stalk (NSS) [46]. Following this breeding strategy and grouping, inbred lines were divided into two divergent heterotic groups according to the different requirements of the parents of hybrids. In Europe, the flint and dent heterotic groups have been widely developed to produce superior hybrids [47]. However, in China, opinions have differed due to the unclear relationships among Chinese maize inbreds. Zhang et al. suggested that 269 inbred lines could be assigned to six heterotic groups based on different methods [48]. Research using 84 parental lines showed that heterotic groups originating from Lancaster, Reid, Tang SPT, Zi330 and E28 in the early 1990s had become Reid, Tem-tropic I, Zi330, Tang SPT and Lancaster, respectively, in the early twenty-first century [49]. Many studies have divided widely used inbred lines into three or more than heterotic groups [50–52], though some reports have indicated that the inbreds found in China can be assigned to two divergence groups. For example, 362 important inbreds from Southwest China were divided into two heterotic groups: temperate and tropical [53]. Additionally, 155 inbred lines were separated into two groups, with seven subgroups, using 82 SSRs [54], and 367 diverse inbreds lines were divided into two heterotic groups, composed of six subgroups [55]. In this study, two divergent heterotic groups, Shaan A and Shaan B, were cultivated through long-term artificial selection based on breeding experience in China and abroad. The population could be divided into 6 subgroups. The Shaan A and Shaan B groups exhibit a clear population structure that can serve as a reference for hybrid breeding and reduce bias in association analysis. The results suggested that the breeding strategy of two divergent heterotic groups has been successful in this population.

The main objective in maize breeding programmes is to increase grain yield [56]. GWAS is an effective tool for identifying genes and dissecting the genetic architecture related to traits, which furthers genetic improvement in crops [18]. In this study, 51 significant SNPs associated with yield were detected across two environments. By searching for candidate genes 150 kb up- and downstream of significant SNPs, 30 genes that were associated with grain yield were identified. Among these genes, Zm00001d027610 and Zm00001d018819 were identified in both environments. Zm00001d027610 encodes a vegetative storage protein. Previous studies have shown that vegetative storage proteins play an important role in the mobilization of amino acids and defence against biotic and abiotic stresses in *Arabidopsis thaliana* and soybean [57, 58]. Another gene (Zm00001d018819) identified in both environments encodes the viviparous-14 protein, which is involved in the biosynthesis of the ABA pathway in maize according to its functional annotation. ABA plays a key role in diverse growth processes [59]. Therefore, viviparous-14 may be involved in determining grain yield by regulating the biosynthesis of ABA. Moreover, the *GBP14* gene (Zm00001d053298), discovered in Yulin, was annotated as a GeBP transcription factor belonging to the DUF573 family, which includes GeBP and GeBP-like proteins as well as storekeeper and storekeeper-like (STKL) transcription factors (http://pfam.xfam.org/family/PF04504.13). In *Arabidopsis,* GeBP and GeBP-like proteins play roles in cytokine hormone pathway regulation [60]. The STK protein may regulate patatin expression in potato tubers [61], and an STKL factor participates in the glucose (Glc) signalling pathway in *Arabidopsis* [62]. Therefore, the candidate gene can likely increase grain yield. Finally, Zm00001d025617, identified in Yangling, encodes grf 2, belonging to the 14–3-3 protein family; this family includes *zmgf14–4* and *zmgf14–6*, which exhibit prominent expression during maize kernel development [63]. The levels of 14–3-3 proteins significantly decrease under salt stress in maize to improve maize adaption [64]. The GF14 protein may play an important role in response to biotic and abiotic stresses in *Arabidopsis* [65]. Hence, we infer that Zm00001d025617 and these genes belonging to the 14–3-3 protein family have similar functions in maize. More work needs to be conducted to verify the functions of these candidate genes.

The genetic improvement of maize yield not only improves yield potential, but also increases stress tolerance [66]. Grain yield is a complex trait and is highly influenced by environmental variation [67]. The experimental locations in this study were Yangling and Yulin, which are both irrigated areas, but one exhibits a sub-humid monsoon climate and the other a semiarid monsoon climate. The different climate and soil conditions resulted in different grain yields, which ranged from 195.11 (Yangling site) to 442.26 kg/mu (Yulin site). Compared to the candidate genes identified in Yulin, the inbreds cultivated in Yangling displayed more stress-related genes in their genome. For example, Zm00001d034563, which encodes dirigent protein (DIR) 4, belongs to the plant DIR family, which has been reported to respond to environmental stress [68]. Additionally, Zm00001d029679 encodes an ethylene-responsive transcription factor (ERF). *AtERF53* increases drought tolerance by facilitating stress-responsive gene expression in *Arabidopsis* [69]. A short GC-box motif has been identified in the ERF genes, which are essential for the response to ethylene, influencing plant growth and development [70]. Zm00001d033834 encodes a GRAS transcription factor. Relevant studies have shown that GRAS family protein play roles in gibberellin (GA) signaling, which not only regulate pant growth and development, but also respond to abiotic stress [71, 72]. These studies indicate that the genes identified in the present study could be involved in responses to biotic and abiotic stresses. The differences in phenotype and genotype observed at the two locations clearly revealed an interaction between the genotype and environment (G × E), suggesting that the population in Yangling suffered worse growth conditions, which is consistent with the uneven rainfall distribution and continuous high temperatures recorded in Yangling. However, some genes that are significantly associated with drought tolerance, such as *ZmNAC111*, *ZmVPP1* and *ZmDREB2A*, were not identified in this population [73–75]; it is possible that these genes were purified through long-term selection in the population.

## Conclusions

In the present study, the genetic diversity, population structure, relationships and association maps related to the grain yield of 126 inbred lines (AM126) selected from the Shaan A and Shaan B groups cultivated at Northwest A&F University were assessed using high-quality SNPs. The MAF and PIC of AM126 were 0.164 and 0.198, respectively. The Shaan B group exhibited a higher MAF and PIC and more unique SNPs than did the Shaan A group. Therefore, the Shaan B group has a broader genetic basis than the Shaan A group. AM126 was divided into 6 subgroups according to genotype analysis and breeding experience, and the inbreds display low relative kinship. GWAS was performed on this population to identify regions associated with grain yield using a population structure and kinship (Q + K) model. A total of 30 candidate genes related to grain yield were identified, among which two were identified in both environments and the others in a single environment. The largest proportion of the genes identified in this study were associated with abiotic stress, which is consistent with the climate conditions. These results illustrate the genetic characteristics of the breeding population and provide favourable alleles related to maize yield, and they can serve as a guideline for hybrid breeding and genetic improvement in the future.

## Additional files

Additional file 1: **Table S1.** Numbers, groups and names of the AM126 lines. (XLSX 14 kb)

Additional file 2: **Table S2.** The grain yield data of the AM126 lines. (XLSX 17 kb)

Additional file 3: The hapmap file of SNP genotyping data of the AM126 lines. (TXT 13628 kb)

Additional file 4: **Table S3.** Pairwise kinship coefficients between AM126 lines calculated with 46,046 SNPs. (XLSX 57 kb)

Additional file 5: **Figure S1.** Heatmap of AM126 based on the kinship matrix computed using 46,046 SNPs. (PDF 135 kb)

Additional file 6: **Table S4.** Co-ancestral membership of AM126 when K = 6. (XLSX 57 kb)

Additional file 7: **Figure S2.** Frequency map of grain yield measured in the samples from Yangling (a) and Yulin (b). (PDF 220 kb)

Additional file 8: **Figure S3.** Picture illustrating LD decay distance in AM126. (PDF 96 kb)

## Abbreviations

ABA: Abscisic acid
AM126: 126 inbred lines
CV: Coefficient of variation
GA: Gibberellin
GAPIT: Genomic Association and Prediction Integrated Tool-R package
GBS: Genotyping-by-sequencing
GWAS: Genome-wide association study
MAF: Minor allele frequency
PIC: Polymorphism information content
SD: Standard deviation
