# Supplementary figures and images for: Genetic characterization of inbred lines from Shaan A and B groups for identifying loci associated with maize grain yield
Source: BMC Genet. 2018 Aug 23;19:63. doi: 10.1186/s12863-018-0669-9 (PMC6108135; doi:10.1186/s12863-018-0669-9)

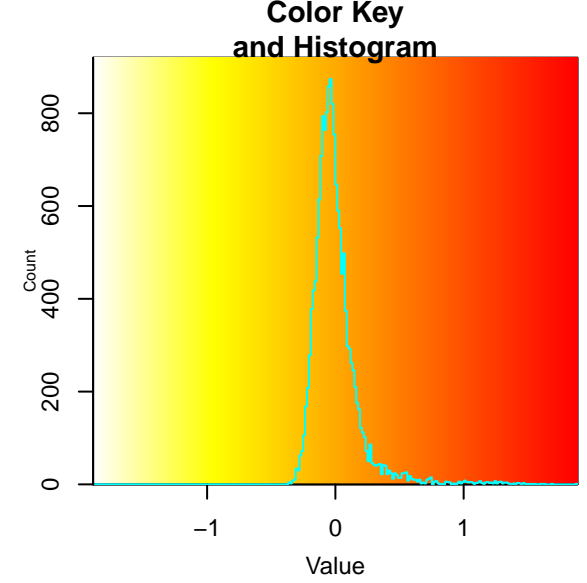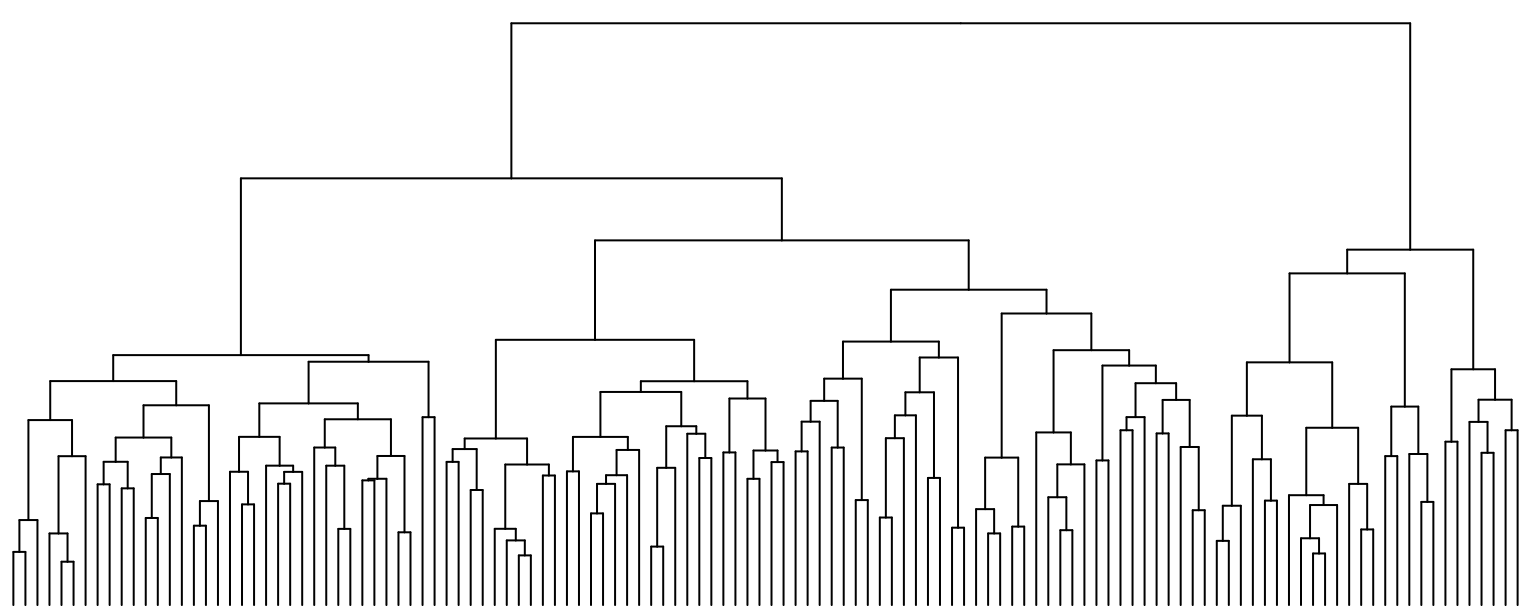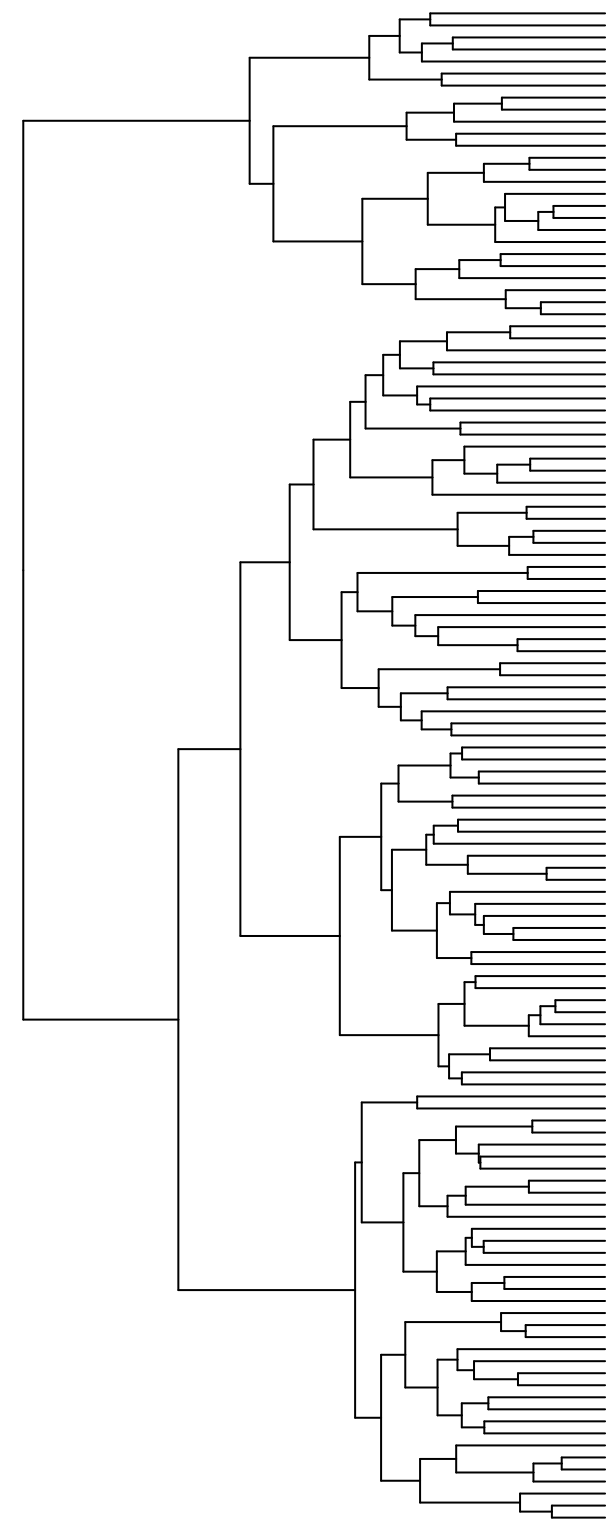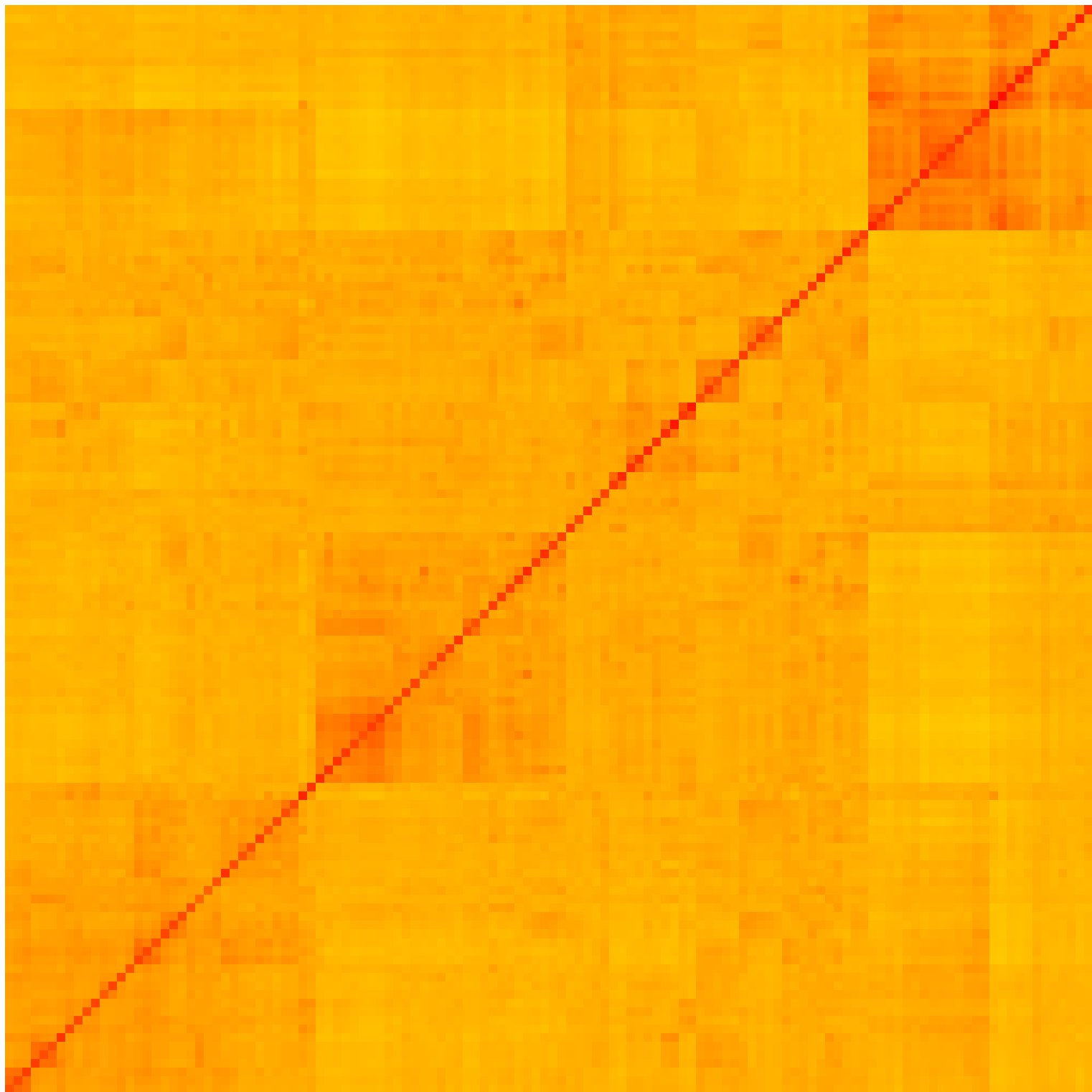

806 811 821 822 823 824 825 826 827 828 829 830 831 832 833 834 835 836 837 838 839 840 841 842 843 844 845 846 847 848 849 850 851 852 853 854 855 856 857 858 859 860 861 862 863 864 865 866 867 868 869 870 871 872 873 874 875 876 877 878 879 880 881 882 883 884 885 886 887 888 889 890 891 892 893 894 895 896 897 898 899 900 901 902 903 904 905 906 907 908 909 910 911 912 913 914 915 916 917 918 919 920 921 922 923 924 925 926 927 928 929 930 931 932 933 934 935 936 937 938 939 940 941 942 943 944 945 946 947 948 949 950 951 952 953 954 955 956 957 958 959 960 961 962 963 964 965 966 967 968 969 970 971 972 973 974 975 976 977 978 979 980 981 982 983 984 985 986 987 988 989 990 991 992 993 994 995 996 997 998 999 1000

Supplement: Supplementary file 5 — Figure S1. Heatmap of AM126 based on the kinship matrix computed using 46,046 SNPs. (PDF 135 kb) [file 12863_2018_669_MOESM5_ESM.pdf]

**a**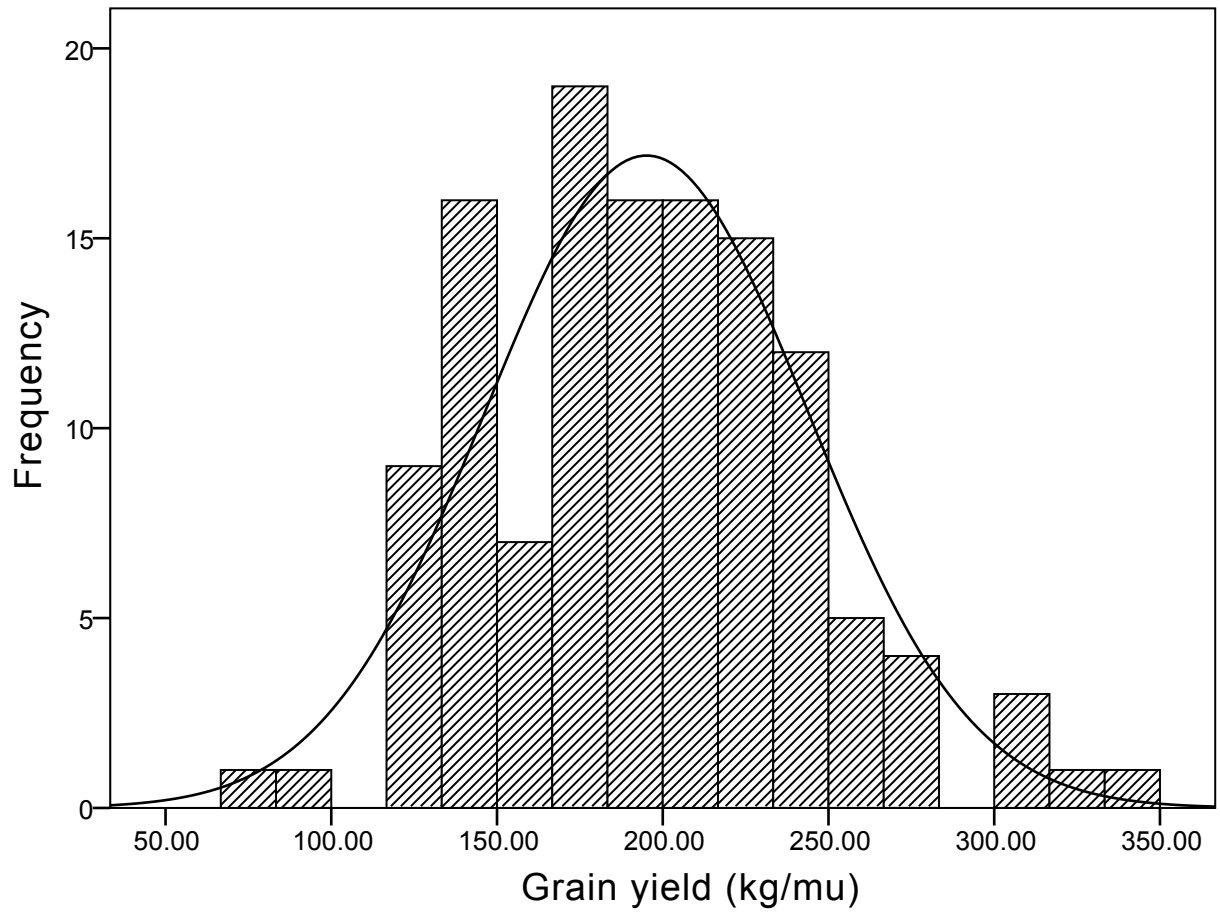**b**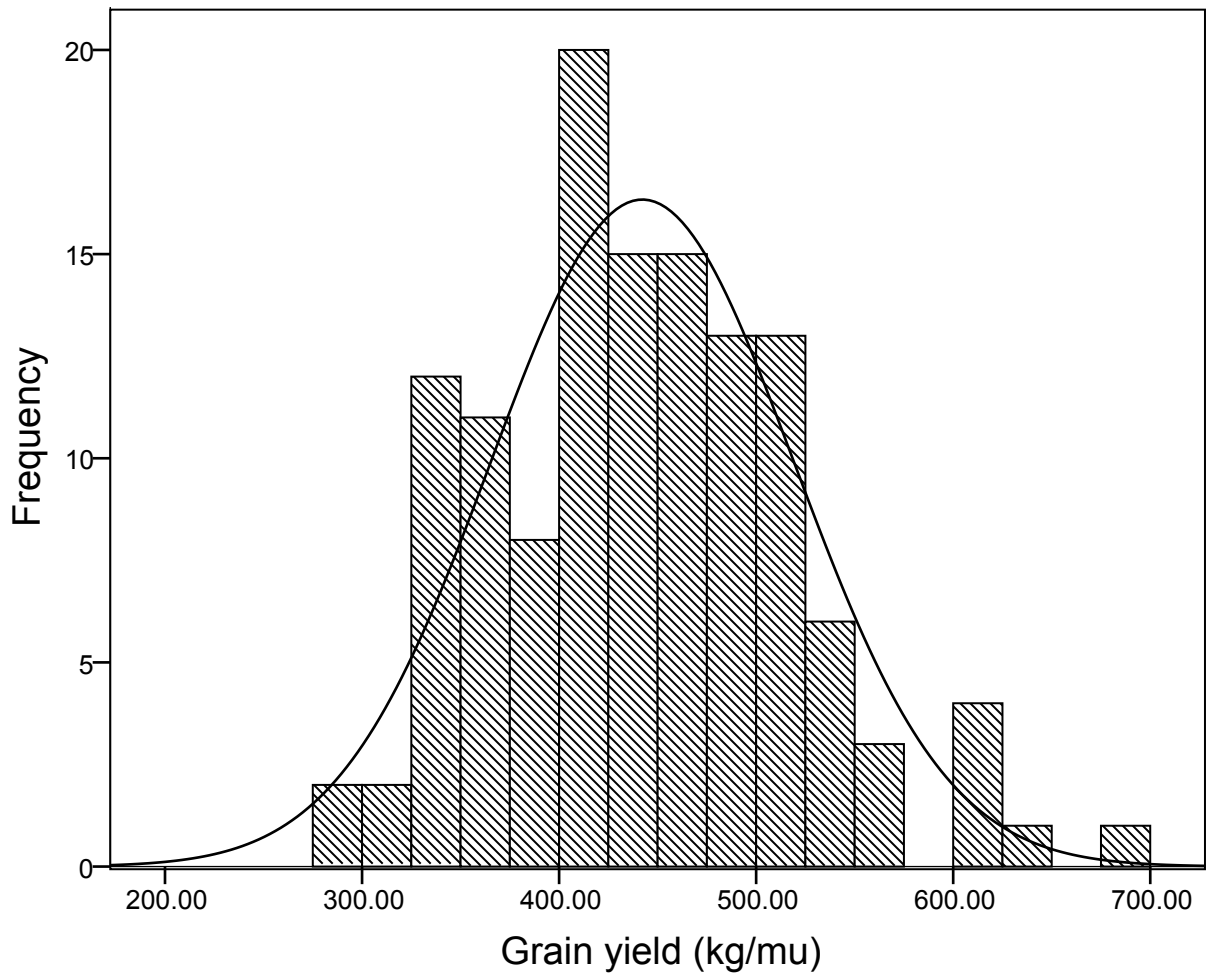

Supplement: Supplementary file 7 — Figure S2. Frequency map of grain yield measured in the samples from Yangling (a) and Yulin (b). (PDF 220 kb) [file 12863_2018_669_MOESM7_ESM.pdf]

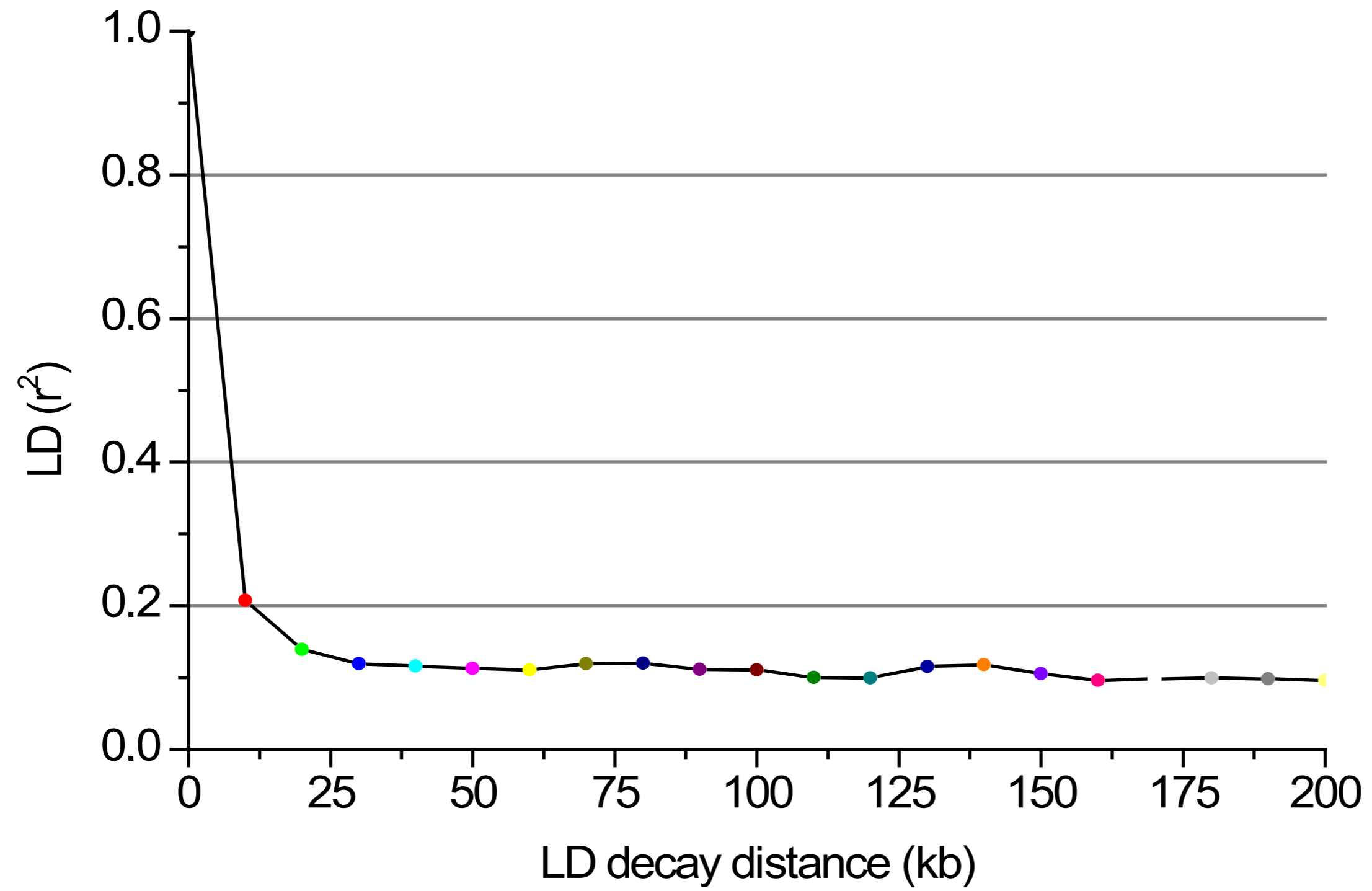

Supplement: Supplementary file 8 — Figure S3. Picture illustrating LD decay distance in AM126. (PDF 96 kb) [file 12863_2018_669_MOESM8_ESM.pdf]
